# Lactate as a major epigenetic carbon source for histone acetylation via nuclear LDH metabolism

**DOI:** 10.1038/s12276-023-01095-w

**Published:** 2023-10-02

**Authors:** Yong Jin An, Sihyang Jo, Jin-Mo Kim, Han Sun Kim, Hyun Young Kim, Sang-Min Jeon, Dawool Han, Jong In Yook, Keon Wook Kang, Sunghyouk Park

**Affiliations:** 1 Natural Products Research Institute, College of Pharmacy, Seoul National University, Seoul, 08826 Korea; 2 College of Pharmacy, Seoul National University, Seoul, 08826 Korea; 3 College of Pharmacy and Institute of Pharmaceutical Science and Technology, Ajou University, Gyeonggi-do, 16499 Korea; 4 Department of Oral Pathology, Oral Cancer Research Institute, Yonsei University College of Dentistry, Seoul, 03722 Korea

**Keywords:** Metabolomics, Epigenetics

## Abstract

Histone acetylation involves the transfer of two-carbon units to the nucleus that are embedded in low-concentration metabolites. We found that lactate, a high-concentration metabolic byproduct, can be a major carbon source for histone acetylation through oxidation-dependent metabolism. Both in cells and in purified nuclei, ^13^C_3_-lactate carbons are incorporated into histone H4 (maximum incorporation: ~60%). In the purified nucleus, this process depends on nucleus-localized lactate dehydrogenase (LDHA), knockout (KO) of which abrogates incorporation. Heterologous expression of nucleus-localized LDHA reverses the KO effect. Lactate itself increases histone acetylation, whereas inhibition of LDHA reduces acetylation. In vitro and in vivo settings exhibit different lactate incorporation patterns, suggesting an influence on the microenvironment. Higher nuclear LDHA localization is observed in pancreatic cancer than in normal tissues, showing disease relevance. Overall, lactate and nuclear LDHA can be major structural and regulatory players in the metabolism–epigenetics axis controlled by the cell’s own status or the environmental status.

## Epigenetics: Lactate implicated in DNA-winding dynamics

Previously regarded as a metabolic byproduct, lactate plays an unexpected role in the modification of DNA-winding proteins called histones. A team led by Sunghyouk Park from Seoul National University, South Korea, showed that lactate serves as a major carbon source for the addition of acetyl groups to histones, an epigenetic modification that alters the coiling of DNA in ways that affect gene expression and can play a crucial role in disease development. The researchers found that lactate, produced during high-energy demand conditions, helps to drive this process with the aid of the enzyme lactate dehydrogenase, which is found in abundance in the nucleus of pancreatic cancer cells. The team’s findings establish a vital link between cellular metabolism and epigenetic regulation, opening potential avenues for targeted anti-cancer therapies that disrupt lactate-mediated acetylation or target the enzyme.

## Introduction

Lactate has long been considered a metabolic waste that is secreted to maintain glycolysis, especially in cancer cells. However, recent studies have suggested that it can be a source of energy in tumor cells^1,2^ or even a signaling molecule^3,4^. More generally, lactate has been suggested to be the primary source of the TCA cycle, even over glucose, in most normal and tumor tissues^5^. These cellular uses of lactate suggest that lactate metabolism can operate in the reverse direction of the well-known formation of lactate from pyruvate. In addition to these bioenergetic roles, large variations in lactate concentrations in different physiological states or tissues indicate that lactate may have structural and regulatory roles sensitive to a cell’s own status or the environmental status.

The nucleus is the largest organelle containing genetic information. Utilization or replication of genetic material requires a large amount of energy and metabolites that are provided mostly by the cytosol^6,7^. Nevertheless, it has also been established that the nucleus has its own versions of metabolic enzymes, including most glycolytic enzymes, to efficiently regulate gene expression according to the cell’s status^8^. The ultimate effects of a particular enzymatic reaction, however, may differ between the cytosol and the nucleus due to the functional differences of the two compartments. For example, acetyl-coenzyme A synthetase 2 (ACSS2) generates acetyl-CoA for fatty acid synthesis in the cytosol, but the same enzymatic reaction is involved in epigenetic histone acetylation in the nucleus^9,10^. In this sense, the functional aspects of many nucleus-present metabolic enzymes have yet to be defined.

Epigenetic histone modification is an important regulatory process in a variety of (patho)physiological conditions. Of note, both the carbon carriers (i.e., Acetyl-CoA and S-adenosylmethionine) and regulatory metabolites (NAD^+^ and oxoglutarate) are quantitatively consumed during modification and regulatory metabolism. Therefore, the concentrations of these metabolites are key factors that connect metabolism and epigenetics^11^. For mammalian histone acetylation, acetyl-CoA is the most immediate carbon donor that, in turn, can be derived from several exogenous carbon sources, such as glucose, acetate, and fatty acids^12,13^. Acetyl-CoA from mitochondria is transported as citrate to the cytosol and is regenerated to acetyl-CoA by adenosine triphosphate (ATP)-citrate lyase (ACLY). However, acetyl-CoA is unstable, and its concentration is low in the cytosol^14^. Paradoxically, fatty acid-driven histone acetylation seems ACLY independent^15^, and acetate usage for histone acetylation occurs mainly in experimentally ACLY-compromised conditions. Therefore, questions remain about the identities of the cytosolic carriers and the ultimate source of the two-carbon unit for histone acetylation under physiological conditions.

Currently, known carbon sources for histone acetylation, such as glucose, acetate, and fatty acids, are also bioenergetic carbon sources. As stated above, lactate is a newly recognized bioenergetic metabolite and can be present at high concentrations both inside and outside cells. Therefore, in the present study, we explored the possibility of lactate being a structural source and regulator of histone acetylation.

## Materials and methods

### Chemicals and reagents

DMEM (no glucose, glutamine, pyruvate, or phenol red) and DMEM (no glucose or pyruvate) were purchased from Gibco, Thermo Fisher (Waltham, MA, USA). Sodium L-lactate-^13^C_3_ solution (45–55% (w/w) in H_2_O, ≥99 atom % ^13^C) from Sigma‒Aldrich (St. Louis, MO, USA) or ^13^C_3_-sodium L-lactate (98%-^13^C_3_, 20% W/W in H_2_O) from Cambridge Isotope Laboratories (Tewksbury, MA, USA) were used interchangeably after correcting the concentration with distilled water. All other isotopically labeled compounds were purchased from Cambridge Isotope Laboratories.

### Cell lines and cultures

The human pancreatic cancer cell line PANC-1 was obtained from the Korean Cell Line Bank (KCLB Cat# 21469) and grown in DMEM (Welgene, Daegu, Korea) supplemented with 10% heat-inactivated fetal bovine serum (FBS, Welgene, Daegu, Korea) and 1% penicillin‒streptomycin solution (Gibco, Thermo Fisher, Waltham, MA, USA). Cells were cultured at 37 °C and 5% CO_2_ in a humidified incubator.

### Intracellular metabolite concentration measurement and tracer metabolism

Cells (1 × 10^8^) were harvested and washed with PBS twice. One milliliter of cold MAD extraction buffer (methanol:acetonitrile:DW; 5:3:2) was added and vortexed for 1 min to extract the intracellular metabolites. The sample was centrifuged at 28,000 × *g* for 20 min at 4 °C, and the supernatant was carefully transferred to a new 1.5 mL tube. The supernatant was then dried with a centrifugal concentrator and kept at −20 °C until further analysis. For analysis of the extent of tracer metabolism, two-phase extraction was employed as follows. Cell plates were washed twice with cold PBS, and cells were transferred to a tube with a scraper. The cells were washed with cold PBS again, spun down, and resuspended in 400 μL of methanol and 200 μL of chloroform. The sample was frozen for 1 min with liquid N_2_, melted at room temperature for 2 min, and mixed for 30 s using a vortex mixer. This process was repeated three times. Then, 200 μL of chloroform and 200 μL of distilled water were added, and the lysates were separated by centrifugation at 15,000 × *g* for 20 min at 4 °C. After centrifugation, the aqueous layer and organic layer were carefully transferred into new 1.5 mL microcentrifuge tubes. The water layer, the organic layer and the pellet were completely dried with a vaccum centrifugal concentrator. The aqueous layer and the organic layer were stored at −20 °C until further analysis. The protein pellet was used for protein normalization.

### Nucleus isolation

All of the experiments were carried out on ice. Adherent cells were washed twice with 10 mL of cold PBS and scraped from the plate with 1 mL of PBS into a 2 mL microtube. The cells were washed with 1 mL of cold hypotonic buffer (10 mM HEPES, 10 mM KCl, 1.5 mM MgCl_2_, 0.3 μM aprotinin, 1.5 μM pepstatin A, 0.5 mM phenylmethylsulfonyl fluoride (PMSF), 1 mM DTT, 1 mM sodium butyrate, and 1 μM trichostatin A), and the buffer was removed by centrifugation at 10,000 rpm for 10 sec. The cells were resuspended in 10 volumes of cold hypotonic buffer containing 0.1% NP-40 and left to swell on ice for 30 min. The cell membrane was gently broken by passages through a 10 mL syringe with a dual nozzle (1 mm diameter and 10 mm length) approximately 10 times, during which the nucleus release was monitored under a microscope. The sample was centrifuged at 800 × *g* at 4 °C for 10 min, and the supernatant (cytosol) was removed. The pellet was resuspended in 1 mL of PBS containing 0.3 μM aprotinin, 1.5 μM pepstatin A, 0.5 mM PMSF, 1 mM DTT, 1 mM sodium butyrate, and 1 μM trichostatin A. The sample (500 µL) was loaded carefully over 2 mL of 30% sucrose in a round-bottomed centrifuge tube and centrifuged at 800 × *g* at 4 °C for 10 min, and the top layer was removed by aspiration. The nuclear pellet was washed with 1 mL of PBS and centrifuged at 800 × *g* at 4 °C for 10 min twice.

### Nuclear metabolism

For nuclear metabolism analysis, medium without pyruvate, glutamine or glucose was used. This medium was supplemented with 10% dialyzed FBS, 10 mM HEPES, 1 mM DTT, 0.3 μM aprotinin, 1.5 μM pepstatin A, 0.5 mM PMSF, 1 mM sodium butyrate, 1 μM trichostatin A, 1 mM coenzyme A, 1 mM glutathione, 1 mM NAD^+^, and 1 U/μL catalase. The isolated nucleus was resuspended in medium (500 μL) with ^13^C_3_-lactate or ^13^C_3_-alanine (Cambridge Isotope Laboratories, Tewksbury, MA, USA) and incubated overnight at 37 °C and 5% CO_2_ in a humidified incubator.

### Histone extraction

Adherent cells or isolated nuclei were incubated with lysis buffer (8 M urea, 10 mM NaCl, 50 mM Tris-HCl, pH 8.0, 2 mM EDTA, and 1 mM DTT) for 20 min at RT. The NaCl concentration was adjusted to 200 mM, and the sample was centrifuged at 18,000 × *g* for 5 min at RT. The chromatin pellet was washed with washing buffer (8 M urea, 200 mM NaCl, 50 mM Tris-HCl, pH 8.0, 2 mM EDTA, and 1 mM DTT) by inverting the tube for 10 min and centrifuged at 18,000 × *g* for 5 min. The wash was repeated until no more protein could be detected in the washing solution by Bradford assay. Histone was extracted from the final pellet by incubating the sample in 1 mL of 0.2 N HCl overnight at 4 °C with inversion. The sample was centrifuged at 18,000 × *g* at 4 °C for 10 min, and the supernatant was precipitated with 35% trichloroacetic acid for 1 hr on ice. The sample was then centrifuged at 18,000 × *g* at 4 °C for 30 min, and the histone pellet was washed twice with 1 mL of cold acetone by centrifugation at 18,000 × *g* at 4 °C for 10 min. The histone pellet was dried and stored at −20 °C until MS analysis.

### Histone acetylation analysis

Histone bands were separated by 15% SDS‒PAGE, and the histone H4 band was cut out with a razor. The gel piece was put into a tube with 1 mL of destaining buffer (200 mM NH_4_HCO_3_, 40% acetonitrile (ACN)), and the tube was inverted at room temperature for 10 min. The procedure was continued, with replacement of the destaining buffer, until the gel was clear. After the addition of 500 µL of 100% ACN to the gel, the gel was dehydrated by inversion at room temperature for 15 min and then dried with a vacuum centrifugal concentrator. After the addition of 60 μL of 10 mM DTT dissolved in 100 mM NH_4_HCO_3_, the sample was incubated at 56 °C for 45 min, and the remaining solvent was removed. Iodoacetamide (60 μL of 55 mM dissolved in 100 mM NH_4_HCO_3_; iodoacetamide treatment was not required in our case but was performed for consistency) was added to the sample, which was shaken and then allowed to stand at room temperature for 30 min. After removing the solvent, the sample was washed with 200 µL of 100% ACN at room temperature for 10 min. Then, the ACN was removed, and the gel was dried using a vacuum centrifugal concentrator. Asp-N (Sigma‒Aldrich, St Louis, MO, USA) was added (0.6 µg per sample), and the sample was incubated overnight at 37 °C. The cleaved peptides were extracted from the gel by inversion for 10 min at room temperature in 200 μL of extraction solution (50% ACN, 0.1% TFA), dried using a vacuum centrifugal concentrator, and stored at −20 °C until analysis with the MALDI TOF-TOF 5800 System (AB SCIEX, Framingham, MA, USA) at the National Center for Inter-University Research Facilities, Seoul National University, Seoul, Korea.

### NMR measurement

For the medium analysis, 450 μL of medium was supplemented with 50 μL of 10x D_2_O NMR buffer (50 mM NaH_2_PO_4_, 20 mM Na_2_HPO_4_, 0.25% TSP). For the extracted metabolites, the aqueous layer was dissolved in 1x D_2_O NMR buffer (5 mM NaH_2_PO_4_, 2 mM Na_2_HPO_4_, 0.025% TSP in 100% D_2_O). The lipid-soluble layer was dissolved in 100% CDCl_3_. The samples were transferred to a 5 mm NMR tube. The 2D-HSQC NMR spectra were obtained using an 800 MHz Bruker Avance spectrometer equipped with a cryogenic triple-resonance probe (Bruker, Billerica, MA, USA). The HSQC spectra were obtained with a Bruker pulse sequence hsqcetgpsisp2.2. The spectral widths were set to 16 ppm (^1^H) and 40 ppm (^13^C), with 1024 (T2) × 120 (T1) complex points and 4 scans per increment.

### Live in-nucleus NMR

For in-nucleus metabolism analysis, 45 μL of medium (pyruvate and glucose-free medium with 10% dialyzed FBS) was supplemented with 5 μL of 10x D_2_O NMR buffer (50 mM NaH_2_PO_4_, 20 mM Na_2_HPO_4_, 0.25% TSP). The isolated nucleus was resuspended in 50 μL of medium with 5 mM ^13^C_3_-lactate (Cambridge Isotope Laboratories, Tewksbury, MA, USA). Then, the mixture was transferred to a 1.7 mm NMR tube that was inserted into a 5 mm NMR tube. The ^1^H-^13^C 2D-HSQC NMR spectra were obtained continuously with 35 cycles for 10 min, as described previously^16^.

### Immunofluorescence

Cells were plated at 3 × 10^5^ cells/well on a confocal dish for 48 hr in a CO_2_ incubator. Two hundred microliters of MitoTracker (Thermo Fisher, Waltham, MA, USA) was added to the cells, which were incubated for 30 min. The cells were then washed twice with cold PBS, fixed with 4% paraformaldehyde for 30 min at RT, and rinsed 3 times for 5 min with PBS. Permeabilization was performed with 0.2% Triton X-100 in PBS for 30 min at RT. Blocking was performed with 2% BSA in PBS for 30 min at RT. Primary antibody incubation (3582S for LDHA, Cell Signaling, Danvers, MA, USA) was performed overnight in 2% BSA in PBS at 4 °C. On the following day, the cells were washed 3 times for 10 min with 2% BSA in PBS at RT and then incubated for 1 hr with goat anti-rabbit IgG H&L (Alexa Fluor 488) (ab150077, Abcam, Cambridge, UK) in PBS at RT. Ten microliters of Hoechst 33342 (Thermo Fisher, Waltham, MA, USA) in PBS was added, and the cells were incubated for 10 min at RT before being washed 3 times for 10 min with 2% BSA in PBS at RT. The coverslip was detached from the confocal dish and mounted on a slide glass with mounting medium. It was then sealed using nail polish and kept at 4 °C. The target protein, mitochondria and cell nuclei were visualized using a TCS8 confocal microscope (Leica Microsystems, Wetzlar, Germany). To quantify LDHA distributed in the cytosol and nucleus, a previously reported method, “Cyt/Nuc”, was used^17^.

### Immunohistochemistry for LDHA

Tissue microarrays of pancreatic adenocarcinoma tissues with matched adjacent normal pancreatic tissues were purchased from US Biomax (PA241d, MD, USA) and re-evaluated by a pathologist (JIY among the authors). As we found that the matched normal tissue in Patient 3 did not include normal pancreatic cells, we excluded Patient 3 from further analysis. The tissues were stained following a standard immunohistochemistry protocol using an antibody for LDHA (3582S, Cell Signaling, Danvers, MA, USA), DAB (3,3′-diaminobenzidine), and hematoxylin. The tissue slides were mounted, and the images were analyzed with IHC profiler^18^. In addition, high-resolution images from the Human Protein Atlas were also used with permission for liver and pancreatic cancer and normal tissues.

### Knockout of LDHA

Cells were plated at 1 × 10^5^ cells/well on a 24-well dish and incubated at 37 °C in a CO_2_ incubator for 24 hr. They were washed twice with medium without serum and 500 µL of medium without FBS, and then antibiotics were added. The cells were starved for 4 hr. Lipofectamine 3000 (Thermo Fisher, Waltham, MA) was used for CRISPR‒Cas9 (SpCas9-2A-Puro V2.0) transfection following the manufacturer’s protocol. After 2 hr of incubation, 10% FBS and 1% penicillin were added. One day later, the medium was replaced with new medium containing 1 μg /mL puromycin (Santa Cruz, Dallas, TX, USA), which was repeated every 48 hr. One week later, the living cells were recovered and separated as individual single colonies, and the knockout of the target protein was confirmed using western blotting. The sequence used for CRISPR‒Cas9 was as follows. LDHA: TGTCATCGAAGACAAATTGAAGG.

### Isotopically labeled lactate administration to animals

After Institutional Animal Ethics Committee approval was obtained at Seoul National University (SNU-191223-1), female BALB/c mice, 10 weeks of age and 19.71 g average weight, were purchased from Orient Bio (Gyeonggi-do, Korea). Before experimentation, the mice were fasted for 4 hr. Twenty or ten milligrams of sodium ^13^C_3_-lactate (Sigma‒Aldrich, Saint Louis, MO, USA) in PBS was injected via the tail vein. The mice were sacrificed at 0, 1, and 4 hr. The liver was washed with saline solution, quickly frozen using liquid nitrogen, and stored at −80 °C until further analysis.

### Bioinformatics analysis

The R package “clusterProfiler” was used for Gene Ontology (GO) overrepresentation analysis and was applied to the GSE29406 dataset. MCF7 cells that were not treated with lactate were chosen as the controls. Only the significantly different genes (*p* value < 0.05), as determined by Student’s *t* test, were drawn on the heatmap. The genes associated with the GO terms were retrieved from Mouse Genome Informatics^19^.

### Western blot analysis

Western blot analysis was performed according to a standard protocol. The following antibodies were used to detect each target protein: anti-LDHA (3582 S, Cell Signaling, Danvers, MA, USA), anti-Lamin A (sc-71481), anti-GAPDH (sc-31915), anti-Cytochrome C (sc-13560), anti-β-actin (sc-47778, Santa Cruz, Dallas, TX, USA), anti-Histone H4 (ab10158, Abcam, Cambridge, UK), anti-acetyl-histone H4 (#06-866, Merck, Kenilworth, NJ, USA), anti-mouse (sc-2005), anti-rabbit (sc-2004), and anti-goat IgG-HRP (sc-2922, Santa Cruz, Dallas, TX, USA) antibodies.

### Chromatin immunoprecipitation sequencing

PANC-1 cells treated with 0 and 10 mM lactate were cross-linked using formaldehyde (final 1%) in culture medium for 10 min at RT and quenched with glycine (final 100 mM) for 5 min at RT. The cell pellet was then resuspended in lysis buffer and sonicated (10 s pulse and 10 s rest, 60 cycles at 60% amplification). Immunoclearing was performed using A/G-agarose beads (sc-2003, Santa Cruz, Dallas, TX, USA) for 2 hr at 4 °C. Immunoprecipitation was carried out overnight at 4 °C using an acetyl-histone H4 antibody (#06-866, Merck), a rabbit IgG antibody (#02-6102, Invitrogen) and A/G-agarose beads. Chromatin was recovered, and RNA and protein were removed using RNase A (IBS-BR003, iNtRON, Gyeonggi-do, Korea) and Proteinase K (E0491, Thermo Fisher, Waltham, MA, USA), respectively. DNA purification was performed manually. The samples were subjected to next-generation sequencing (Illumina NovaSeq sequencing (100 bp paired-end, 10 Gb) at Macrogen, Inc. (Seoul, Korea). The data have been deposited in NCBI’s Gene Expression Omnibus and are accessible through GEO Series accession number GSE235471.

## Results

### Lactate as a carbon source for N-acetylation

**Fig. 1:**
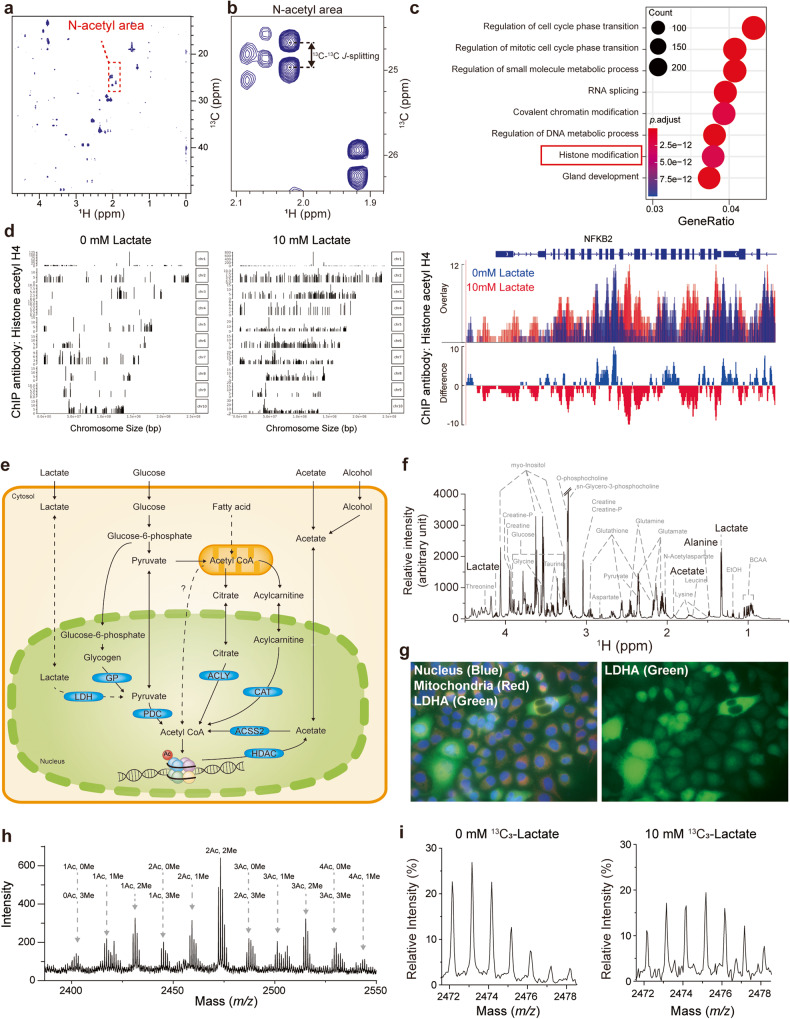
Cellular lactate metabolism provides carbons for histone acetylation. **a** ^1^H-^13^C 2D HSQC spectrum for the extract of PANC-1 cells treated with 10 mM ^13^C_3_-lactate. The dashed box corresponds to the acetyl signals from N-acetyl compounds. **b** ^13^C-^13^C *J*-splitting of acetyl signals of N-acetyl compounds. **c** Gene Ontology analysis of genes affected by 10 mM lactate treatment in MCF-7 cells. **d** Differences in ChIP peaks of histone acetyl H4 on chromosomes following 0 or 10 mM lactate treatment in PANC-1 cells (left panel). Difference in the ChIP peak of histone acetyl H4 at the NFKB2 gene region, chr10:102,392,109-102,404,529 (right panel). 0 mM lactate: blue; 10 mM lactate: red; gray box: promoter region. **e** External carbon sources for histone acetylation and related metabolism: lactate (current study) and others from the literature. CAT carnitine acetyltransferase, GP glycogen phosphorylase, HDAC histone deacetylases, PDC pyruvate dehydrogenase complex. **f** Intracellular metabolites of PANC-1 cells as observed by ^1^H-NMR. PANC-1 cells were grown in 100 pi dishes with DMEM, and metabolites were extracted using chloroform, methanol, and DW. Metabolite concentrations were obtained from the integration of these peaks with Chenomx software (Edmonton, Canada). **g** Nuclear localization of LDHA in PANC-1 cells as visualized by immunofluorescence. The nucleus was stained with Hoechst (blue), mitochondria with MitoTracker (red), and LDHA with an antibody conjugated with Alexa 488 (green). **h** MALDI-TOF spectrum of the histone H4 tail generated by Asp-N digestion of histones from PANC-1 cells. Peak clusters for different combinations of methylation and acetylation are indicated. **i** 2Ac, 2Me cluster on H4 MALDI-TOF spectrum from PANC-1 treated with 0 or 10 mM ^13^C_3_-lactate. The spectra were normalized according to the total intensities within each cluster.

To obtain insights into the metabolic fate of lactate in cells, we treated ^13^C-labeled lactate to cells (PANC-1) and profiled the ^13^C-labeled metabolites with ^1^H-^13^C 2D HSQC NMR-based metabolomics. Metabolites derived from the added ^13^C_3_-lactate were identified based on the presence of ^13^C-^13^C *J*-splitting^20^, and they included acetate, alanine, glutamate, glutathione, aspartate, proline, and N-acetyl aspartate (Fig. 1a and Supplementary Fig. 1a). Most of these metabolites could be explained by known biosynthetic or bioenergetic metabolism for either the C2 or C3 units from lactate. Among them, peaks for N-acetyl aspartate and other N-acetylated amino acids in the regions of 1.8–2.1 ppm (^1^H) and 22–28 ppm (^13^C) drew our attention (Fig. 1b). N-acetylation is a functional modification, as in histone acetylation or N-terminal acetylation, rather than a bioenergetic or biosynthetic process. Therefore, we hypothesized that lactate carbons might be incorporated into functional targets.

To identify the functional effects of external lactate, we studied the effects of lactate on gene expression profiles. Gene Ontology enrichment analysis of a microarray dataset (GSE29406) identified various gene sets affected by lactate treatment (Fig. 1c and Supplementary Fig. 1b). Notably, the Gene Ontology terms for “histone modification” and “protein localization to nucleus” were among those with the highest significance. Moreover, ChIP-seq analysis revealed an overall increase in histone H4 acetylation on chromosomes in response to external lactate (Fig. 1d and Supplementary Table 1). Representatively, extensive acetylation of histone H4 was confirmed at the chromosomal region (including the promoter region) of nuclear factor kappa B subunit 2 (NFKB2), which is known as a chromatin-modifying enzyme (promoter ≤1 kb, 1-2 kb, and exon 38) (Fig. 1d). These results support the idea that external lactate provides carbons for N-acetylation and modulates histone acetylation.

### Intracellular lactate concentration and the presence of LDHA in the nucleus

Currently known two-carbon carriers for histone acetylation, such as pyruvate, citrate, and acetate, diffuse from the cytosol into the nucleus and are converted to acetyl-CoA in situ to generate nuclear acetyl-CoA (Fig. 1e). Despite the large sizes of nuclear pores, there seem to be barriers to free passage of metabolites between the cytosolic and nuclear compartments^7^. Therefore, a C2 source with the highest cytosolic concentration should be best suited for efficient diffusion to dynamically regulate epigenetic modification. Measurement of the intracellular concentrations of these C2 sources in PANC-1 cells by ^1^H-NMR (Fig. 1f and Supplementary Table 2) showed that the lactate concentration (2.22 pM/cell) was approximately an order of magnitude higher than those of acetate (0.09 pM/cell) and pyruvate (0.25 pM/cell) and that the citrate concentration was under our detection limit. Another factor necessary for nuclear metabolism of a C2 source is the presence of the relevant enzyme in the nucleus. For lactate to be used as an acetylation source, it must be converted to acetyl-CoA via pyruvate, which is successively mediated by LDH and pyruvate dehydrogenase (PDH). As PDH is known to be present in the nucleus^21–23^, we checked for the presence of LDH therein. Immunocytochemical staining of the cells showed that LDHA was broadly dispersed within the cells, 65% in the cytosol and 35% in the nucleus (Fig. 1g). Overall, the findings indicate that lactate may diffuse into the nuclear compartment, where it can be converted to acetyl-CoA via LDH and PDH.

### Lactate as a source of acetyl groups for histone acetylation

To confirm the actual involvement of lactate in histone acetylation, we investigated the incorporation of ^13^C-lactate carbons into acetylated histones, particularly H4. For this, we exploited an enzyme called Asp-N that can cleave the N-terminal 23-residue tail peptide of H4, which has 6 acetylation sites (R3, K5, K8, K12, K16, and K20; Supplementary Fig. 2a). The MALDI-TOF spectrum of the Asp-N digest of H4 from the cells treated with ^13^C_3_-lactate (10 mM) showed clusters of peaks for various combinations of H4 tail modifications (Fig. 1h). Among them, the modification with diacetylation and dimethylation (2Ac2Me) was the most prominent (*m/z* = 2472 ~ 2479) and thus was used for further analysis. Detailed isotopomer analysis of the peaks in that cluster showed a shift of the isotopomer peak profile by *m/z* = 2 between the control and ^13^C_3_-lactate-treated groups (Fig. 1i and Supplementary Fig. 2b), confirming the incorporation of the ^13^C_2_-acetyl group derived from ^13^C_3_-lactate into histones. In addition, the incorporation ratio of ^13^C_3_-lactate for this particular modification was ~55%, which showed that lactate can be a major carbon source for histone acetylation. Overall, our results show that lactate can not only be oxidized as an energy source but also be a direct carbon source for the functional modification of histones.

### Lactate-to-pyruvate conversion through nucleus-inherent metabolism

**Fig. 2:**
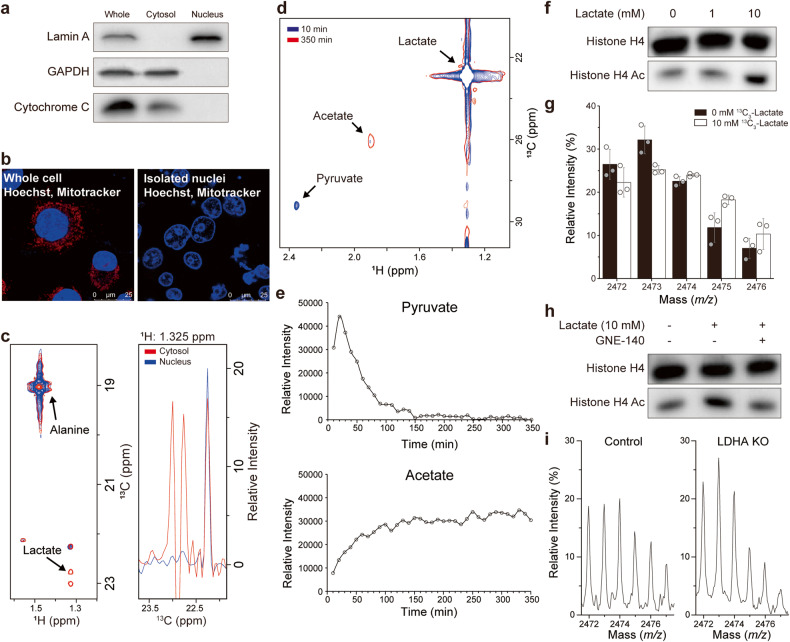
Lactate incorporation into H4 in isolated nuclei by nuclear LDHA. **a** Analysis of the purity of isolated nuclei by western blotting. Lamin A was used as a nuclear marker, GAPDH was used as a cytosolic marker, and Cytochrome C was used as a mitochondrial marker. **b** Purity and membrane integrity of isolated nuclei by immunofluorescence. The nucleus was stained with Hoechst (blue), and the mitochondria were stained with MitoTracker (red). **c** Test of cytosolic contamination with cytosolic alanine transaminase activity. Left, overlay of ^1^H-^13^C 2D HSQC spectra for separated cytosol (red) and nuclear (blue) fractions after addition of ^13^C_3_-alanine. Right, 1D slice of the left 2D spectra along the lactate peak at ^1^H = 1.325 ppm. **d** Metabolism of ^13^C_3_-lactate in isolated nuclei as observed with 35 cycles of 10 min by ^1^H-^13^C 2D HSQC. The figure shows the spectra of the first cycle (blue) and the last cycle (red). **e** Real-time metabolism of ^13^C_3_-lactate in isolated nuclei monitored with in-nucleus NMR^16^. The graphs represent the peak intensities for pyruvate (upper) and acetate (lower), hence the time-dependent metabolite changes. **f** Concentration-dependent effects of lactate on H4 histone acetylation in isolated nuclei. **g** *M/z* values of the MALDI-TOF spectrum of the Asp-N digest of H4 obtained from isolated nuclei treated with 0 (filled bars) or 10 mM (empty bars) ^13^C_3_-lactate. The plot is for the 2-methylation and 2-acetylation cluster normalized to the sum of the total intensities of each group. **h** Effects of GNE-140 (100 μM), an LDH inhibitor, on histone acetylation in isolated nuclei. **i** Representative MALDI-TOF spectra of the Asp-N digest of histone H4 for isolated nuclei from control and LDHA-KO PANC-1 cells treated with 10 mM ^13^C_3_-lactate (same cluster and normalization as in **g**).

Next, to confirm that lactate can be converted to pyruvate in the nucleus for histone acetylation, we obtained a highly purified nuclear fraction without cytosolic and mitochondrial contamination, judged by compartment-specific markers (Fig. 2a). The fluorescence image also showed intact membranous structures of the isolated nucleus (Fig. 2b). Furthermore, the conversion of ^13^C_3_-alanine to ^13^C_3_-lactate, which requires cytosolic alanine aminotransferase, was observed in the cytosolic fraction but not in the nuclear fraction, confirming the absence of observable cytosolic enzymatic activities (Fig. 2c). We also set up a reaction mixture with catalase to suppress the nonenzymatic conversion of pyruvate to acetate reported previously (Supplementary Fig. 3a, b)^24,25^. To the thus-prepared purified nucleus, ^13^C_3_-lactate was added, and the metabolites derived from it were analyzed with ^1^H-^13^C 2D HSQC NMR. ^13^C-methyl peaks for pyruvate and acetate were observed (Fig. 2d), confirming lactate-to-pyruvate conversion in the purified nucleus. Interestingly, the peak intensities were opposite for acetate and pyruvate at the two time points, suggesting different time dependences for these metabolites.

Therefore, we monitored the real-time concentration changes for acetate and pyruvate generated from nuclear lactate metabolism using live NMR metabolomics (Fig. 2e)^26,27^. The pyruvate concentration peaked at a very early time point, after which it steadily decreased. In contrast, the acetate concentration gradually increased. This stark difference indicates that pyruvate is an early intermediate that is rapidly consumed upon its generation from lactate, whereas acetate is an end-product accumulating to a substantial degree in this time frame. These results can be explained by the nuclear LDH-mediated conversion of lactate to a pyruvate intermediate that is subsequently converted to acetyl-CoA or acetate by PDH. The latter reaction was demonstrated recently^28^.

Next, we investigated whether nuclear lactate metabolism actually increases histone acetylation. Lactate treatment of the isolated nucleus increased H4 acetylation in a concentration-dependent manner (Fig. 2f). ^13^C-lactate treated to isolated nuclei was also incorporated into histones, as shown by MALDI-TOF isotopomer analysis (Fig. 2g). In addition, GNE-140, a specific inhibitor of LDH, reversed the increase in H4 acetylation induced by lactate treatment in the isolated nucleus (Fig. 2h). Further MALDI-TOF analysis of isolated nuclei from LDHA-KO cells treated with ^13^C-lactate showed that the absence of LDHA abrogated the incorporation of ^13^C_3_-lactate into H4 (Fig. 2i and Supplementary Fig. 3c). These data show that cytosolic lactate enters the nucleus, is metabolized in situ via nuclear LDHA, and increases histone acetylation by providing the C2 group.

### Lactate’s contribution to acetylation in vivo and association with human cancer

**Fig. 3:**
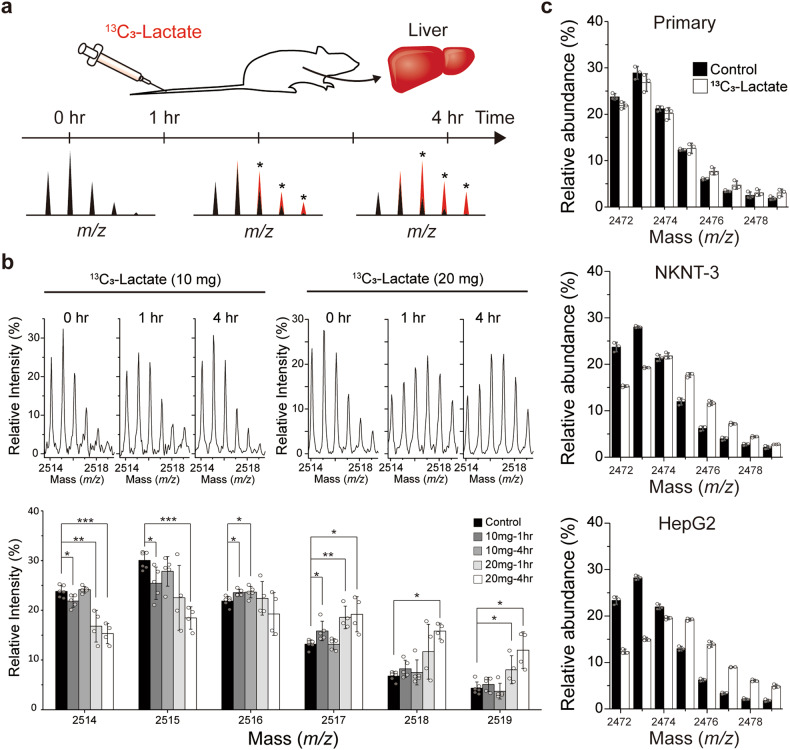
Lactate incorporation into H4 in the mouse liver. **a** Strategies for the in vivo labeling experiment with circulating lactate. ^13^C_3_-lactate at doses of 0.5 and 1 g/kg was injected into the tail veins, and the mice were sacrificed at 0, 1, and 4 hr. Liver tissues were obtained and analyzed for ^13^C-labeled histone with MALDI-TOF. The schematic spectra at the bottom represent the expected patterns. **b** Representative MALDI-TOF spectra for the Asp-N-digested H4 tail obtained from mouse liver. The cluster of 2-methylated, 3-acetylated histone H4 and normalization are as in Fig. 2g. The bottom graphs are for the peak intensities from the biological replicates (*n* = 6 for control, 5 for 10 mg groups, and 4 for 20 mg groups), *p < 0.05, **p < 0.005, ***p < 0.0005 (Student’s *t* test). **c** Acetylation status of the histone H4 tail in different liver cell lines treated with ^13^C_3_-lactate (10 mM). H4 incorporation was analyzed according to the MALDI-TOF spectrum. The cluster of 2-methylated, 2-acetylated histone H4 was normalized by the sum of its total intensities (*n* = 3).

To date, studies on metabolic sources of histone acetylation have been performed mostly with in vitro cell culture systems^15,29,30^. To test the effects of the microenvironment on lactate incorporation into histones, mice were injected with ^13^C_3_-lactate, and their liver tissue was analyzed with the MALDI-TOF system (Fig. 3a). Lactate incorporation was visible at the 1- and 4-hr time points at the two doses, and the higher dose led to more pronounced incorporation, with the maximum incorporation being ~37% (Fig. 3b and Supplementary Table 3). This result confirms the in vivo relevance of lactate as a carbon source for histone acetylation. Interestingly, the tissue samples and in vitro primary hepatocyte samples exhibited different histone modification profiles: the proportions of the 3Ac2Me and 4Ac2Me clusters were much higher in the tissue samples (Supplementary Fig. 3d). This suggests that epigenetic modification may be affected by in vitro vs. in vivo conditions such as microenvironmental differences. It was also observed that lactate incorporation was higher in the transformed or liver cancer cell lines (NKNT3 or HepG2, maximum ~60%) that proliferate more rapidly than in the primary liver hepatocytes (maximum ~16%; Fig. 3c, and Supplementary Table 4). The acetylation level of histone H4 was also observed to exhibit the same trend (Supplementary Fig. 3e).

**Fig. 4:**
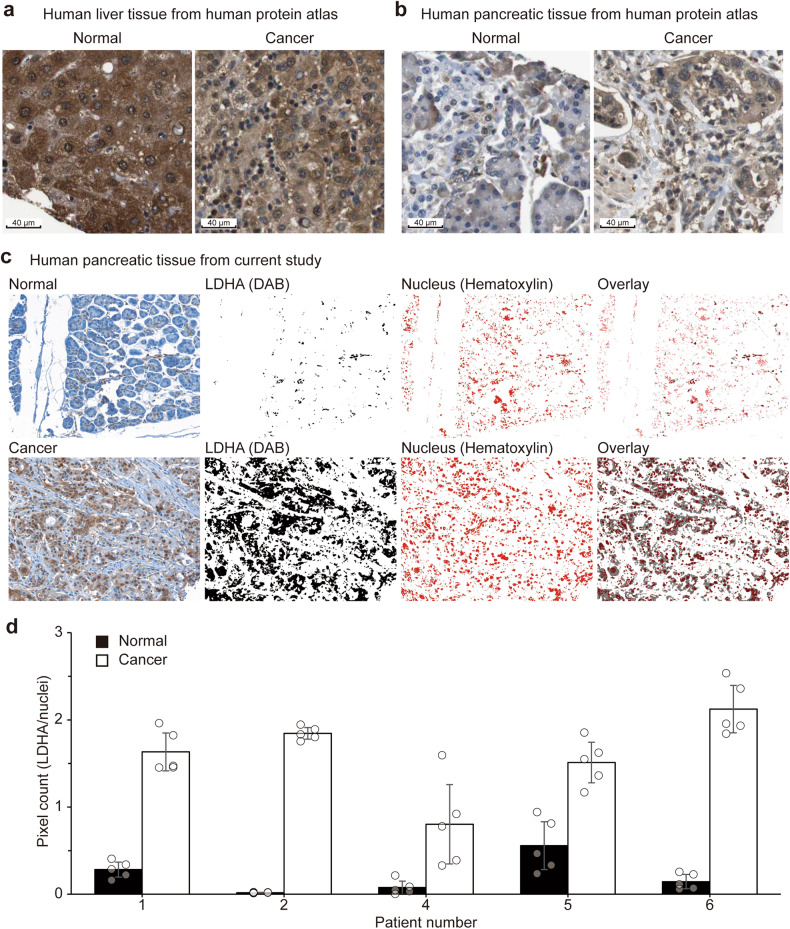
Differential nuclear localization of LDHA in human cancer and normal tissues. Immunohistochemical staining of LDHA in human normal tissues (left) and cancer tissues (right). **a** Liver tissues from the Human Protein Atlas. **b** Pancreatic tissues from the Human Protein Atlas. **c** Representative images from the patient samples that were stained in the current study (*n* = 5). Upper: normal tissues; Lower: cancer tissues. Left-most: Bright field images. From the second-left to right: IHC Profiler analysis of LDHA level (DAB), nuclear space (hematoxylin), and overlay of DAB and hematoxylin. **d** Quantification of the nuclear localization of LDHA with IHC Profiler for the images in **c**. Filled bars are for normal tissues, and empty bars are for cancer tissues. Five random areas (empty circles) were chosen from the images in **c**, and the pixel intensities were analyzed. The error bars indicate the standard deviations.

To determine if this lactate-usage difference is also relevant to human diseases involving rapidly proliferating cells, the presence of LDHA in the nuclear compartment was studied in human normal and cancer tissues (Fig. 4 and Supplementary Table 5). First, for liver tissues in the Human Protein Atlas, despite high LDHA staining in both the cytosol and the nucleus (Fig. 4a), some tumor tissues exhibited higher nuclear staining than normal tissues, especially with the antibody CAB069404 (Supplementary Table 5). For pancreatic tissues in the Human Protein Atlas, the difference was much clearer (Fig. 4b and Supplementary Table 5). Normal pancreatic tissues minimally expressed LDHA, whereas most of the pancreatic cancer tissues exhibited high expression with more nuclear localization of LDHA. To independently confirm this, we stained clinical pancreatic adenocarcinoma tissue arrays that also had matched adjacent normal pancreatic tissues (Fig. 4c and Supplementary Fig. 3f). Unbiased quantification of LDHA abundance using IHC Profiler software^18^ revealed that cancer tissues express more LDHA than normal tissues at the overall cell level (Fig. 4c). More importantly, much higher LDHA abundance was observed in the nuclear space of the cancer tissues than in that of the normal tissues (Fig. 4d). Combined with our in vitro results obtained with the pancreatic cancer cell line PANC-1, these results suggest that lactate incorporation into histones through nuclear LDHA may have implications for pancreatic cancer tumorigenesis. Overall, lactate is used for histone acetylation in vivo, and the process may be sensitive to various cellular or environmental variables, such as transformational status.

## Discussion

Our results describe a new metabolism–epigenetics axis wherein external lactate is metabolized in the nucleus to be used as a carbon substrate for histone acetylation, essentially providing a structural component of chromatin. These findings expand the roles of lactate to a bona-fide structural acetylation source and an epigenetic regulator. In addition, the usage of external lactate in the nucleus is consistent with the lactate shuttle theory that emphasizes lactate oxidation to pyruvate and intercellular/interorganellar lactate transport^31^. The current lactate shuttle theory assumes that mitochondria are major organelles for lactate oxidation, and our results add the nucleus as another candidate compartment. As the equilibrium constant for lactate oxidation is quite small (~1 × 10^−4^)^32^, there must be a driving force for the reaction to occur. Our real-time data show that pyruvate generated from lactate is rapidly consumed in the isolated nucleus. Therefore, the ultimate disposal of lactate-derived pyruvate into histone acetyl groups should be the main driving force for lactate-to-pyruvate conversion in the nucleus. A recent report has also suggested that lactate, as a whole without oxidation, can be attached to histones through lactylation, revealing a nonmetabolic function of lactate in epigenetic control^33^. In contrast, lactate’s involvement in histone acetylation requires oxidation by LDH. It seems that lactate carbons can be used for epigenetics in a dual manner (i.e., oxidation-dependent acetylation or oxidation-independent lactylation). Furthermore, the series of metabolic processes that generate acetyl-CoA from lactate through pyruvate not only provide an essential source for histone acetylation but also increase NADH, which inhibits the activity of SIRT1, a histone deacetylase^34^. During the submission of this manuscript, Torrini C. et al. reported that lactate is used as a direct acetylation carbon source for various histones in glioblastomas, which is consistent with our data^35^. Furthermore, our data reveal that this response is driven by nuclear LDH. Overall, lactate is suggested to be at the crossroads between metabolism and gene regulation.

In our study, external lactate contributed anywhere between 4 and 60% of the acetyl groups of histones depending upon the cells/tissues (see Supplementary Tables 3 and 4). The incorporation ratios also differed substantially among clusters within a single cell line (i.e., 14.8% for 2Me1Ac and 59.7% for 2Me3Ac in HepG2, see Supplementary Table 4). The variability across cells and individual clusters suggests that the lactate contribution is widely tunable according to a cell’s own status or the environmental conditions to allow finer control of lactate-driven epigenetic regulation. It should also be noted that the variability could be due to the different acetylation turnover rates rather than different carbon sources, which should be considered in future research. To date, glucose-derived acetyl-CoA, carried as citrate in the cytosol, has been considered a major carbon source of nuclear histone acetylation^12,29^. Recent studies have also suggested medium-chain fatty acids (MCFAs)^15^ and acetate^36^ as alternative contributors. These results suggest that there should be metabolic plasticity in terms of the structural sources and carriers of histone acetyl carbons.

It is well known that increased histone acetylation generally promotes cell cycle progression and proliferation^8^. In that sense, it is interesting to note that nuclear LDHA is more prevalent in human pancreatic cancer tissue than in normal tissue and that higher LDHA levels are associated with poorer survival prognosis of pancreatic cancer patients (Supplementary Table 6). Consistently, we observed higher histone acetylation in transformed liver cell lines than in normal primary hepatic cells. In addition, a higher level of nuclear LDH is correlated with poor survival in esophageal squamous cell carcinoma, and lactate exhibits profound effects on cancer immune evasion and metastasis^37,38^. Furthermore, nuclear LDHA levels were higher in tumor tissues than in normal tissues from various organs (Supplementary Table 7) and different for various cell lines of the same tumor (Supplementary Fig. 4). These biological phenomena have been mostly explained in terms of the metabolic roles of lactate and LDH. It will be very intriguing to study the detailed roles of lactate-driven histone acetylation in these processes.

### Supplementary information

Supplemental Information

Supplemental Table 1

## References

[1] Faubert B (2017). Lactate metabolism in human lung tumors. Cell.

[2] Kennedy KM (2013). Catabolism of exogenous lactate reveals it as a legitimate metabolic substrate in breast cancer. PLoS ONE.

[3] Lee DC (2015). A lactate-induced response to hypoxia. Cell.

[4] Roland CL (2014). Cell surface lactate receptor GPR81 is crucial for cancer cell survival. Cancer Res..

[5] Hui S (2017). Glucose feeds the TCA cycle via circulating lactate. Nature.

[6] Wright RHG (2016). ADP-ribose-derived nuclear ATP synthesis by NUDIX5 is required for chromatin remodeling. Science.

[7] Diehl KL, Muir TW (2020). Chromatin as a key consumer in the metabolite economy. Nat. Chem. Biol..

[8] Boukouris AE, Zervopoulos SD, Michelakis ED (2016). Metabolic enzymes moonlighting in the nucleus: metabolic regulation of gene transcription. Trends Biochem. Sci..

[9] Mews P (2017). Acetyl-CoA synthetase regulates histone acetylation and hippocampal memory. Nature.

[10] Bulusu V (2017). Acetate recapturing by nuclear acetyl-CoA synthetase 2 prevents loss of histone acetylation during oxygen and serum limitation. Cell Rep..

[11] Su X, Wellen KE, Rabinowitz JD (2016). Metabolic control of methylation and acetylation. Curr. Opin. Chem. Biol..

[12] Sebastian C, Mostoslavsky R (2017). The various metabolic sources of histone acetylation. Trends Endocrinol. Metab..

[13] Feron O (2019). The many metabolic sources of acetyl-CoA to support histone acetylation and influence cancer progression. Ann. Transl. Med..

[14] Pietrocola F, Galluzzi L, Bravo-San Pedro JM, Madeo F, Kroemer G (2015). Acetyl coenzyme A: a central metabolite and second messenger. Cell Metab..

[15] McDonnell E (2016). Lipids reprogram metabolism to become a major carbon source for histone acetylation. Cell Rep..

[16] Xu WJ (2018). Observation of acetyl phosphate formation in mammalian mitochondria using real-time in-organelle NMR metabolomics. Proc. Natl. Acad. Sci. USA.

[17] Grune T, Kehm R, Hohn A, Jung T (2018). “Cyt/Nuc,” a customizable and documenting imagej macro for evaluation of protein distributions between cytosol and nucleus. Biotechnol. J..

[18] Varghese F, Bukhari AB, Malhotra R, De A (2014). IHC profiler: an open source plugin for the quantitative evaluation and automated scoring of immunohistochemistry images of human tissue samples. PLoS ONE.

[19] Yu G, Wang LG, Han Y, He QY (2012). ClusterProfiler: an R package for comparing biological themes among gene clusters. OMICS.

[20] Lee S (2017). Carbon isotopomer analysis with non-unifom sampling HSQC NMR for cell extract and live cell metabolomics studies. Anal. Chem..

[21] Sutendra G (2014). A nuclear pyruvate dehydrogenase complex is important for the generation of acetyl-CoA and histone acetylation. Cell.

[22] Li W (2022). Nuclear localization of mitochondrial TCA cycle enzymes modulates pluripotency via histone acetylation. Nat. Commun..

[23] Zervopoulos SD (2022). MFN2-driven mitochondria-to-nucleus tethering allows a non-canonical nuclear entry pathway of the mitochondrial pyruvate dehydrogenase complex. Mol. Cell.

[24] Cavallini D (1951). The coupled oxidation of pyruvate with glutathione and cysteine. Biochem. J..

[25] Constantopoulos G, Barranger JA (1983). Nonenzymatic decarboxylation of pyruvate. Anal. Biochem..

[26] Wen H, An YJ, Xu WJ, Kang KW, Park S (2015). Real-time monitoring of cancer cell metabolism and effects of an anticancer agent using 2D in-cell NMR spectroscopy. Angew. Chem. Int. Ed. Engl..

[27] Jin X, Kang S, Tanaka S, Park S (2016). Monitoring the glutathione redox reaction in living human cells by combining metabolic labeling with heteronuclear NMR. Angew. Chem. Int. Ed. Engl..

[28] Bose S, Ramesh V, Locasale JW (2019). Acetate metabolism in physiology, cancer, and beyond. Trends Cell Biol..

[29] Wellen KE (2009). ATP-citrate lyase links cellular metabolism to histone acetylation. Science.

[30] Latham T (2012). Lactate, a product of glycolytic metabolism, inhibits histone deacetylase activity and promotes changes in gene expression. Nucleic Acids Res..

[31] Brooks GA (2018). The science and translation of lactate shuttle theory. Cell Metab..

[32] Williamson DH, Lund P, Krebs HA (1967). The redox state of free nicotinamide-adenine dinucleotide in the cytoplasm and mitochondria of rat liver. Biochem. J..

[33] Zhang D (2019). Metabolic regulation of gene expression by histone lactylation. Nature.

[34] Levine DC (2021). NADH inhibition of SIRT1 links energy state to transcription during time-restricted feeding. Nat. Metab..

[35] Torrini C (2022). Lactate is an epigenetic metabolite that drives survival in model systems of glioblastoma. Mol. Cell.

[36] Zhao S (2016). ATP-citrate lyase controls a glucose-to-acetate metabolic switch. Cell Rep..

[37] Brand A (2016). LDHA-associated lactic acid production blunts tumor immunosurveillance by T and NK cells. Cell Metab..

[38] Goetze K, Walenta S, Ksiazkiewicz M, Kunz-Schughart LA, Mueller-Klieser W (2011). Lactate enhances motility of tumor cells and inhibits monocyte migration and cytokine release. Int. J. Oncol..

