# Supplementary material for: Lactate as a major epigenetic carbon source for histone acetylation via nuclear LDH metabolism
Source: Exp Mol Med. 2023 Oct 2;55(10):2238–47. doi: 10.1038/s12276-023-01095-w (PMC10618192; doi:10.1038/s12276-023-01095-w)
Supplement: Supplementary file 1 — Supplemental Information [file 12276_2023_1095_MOESM1_ESM.docx]

**Supplementary Information**

**Lactate as a major epigenetic carbon source for histone acetylation via nuclear LDH metabolism**

Yong Jin An^1^, Sihyang Jo^1^, Jin-Mo Kim^1^, Han Sun Kim^1^, Hyun Young Kim^2^, Sang-Min Jeon^2,3^, Dawool Han^4^, Jong In Yook^4^, Keon Wook Kang^2^, Sunghyouk Park^1, *^

^1^Natural Products Research Institute, College of Pharmacy, Seoul National University, Seoul 08826, Korea

^2^College of Pharmacy, Seoul National University, Seoul 08826, Korea

^3^College of Pharmacy and Institute of Pharmaceutical Science and Technology, Ajou University, Gyeonggi-do 16499, Korea

^4^Department of Oral Pathology, Oral Cancer Research Institute, Yonsei University College of Dentistry, Seoul 03722, Korea

*Corresponding author:

Sunghyouk Park

Phone) +82-2-880-7831; Fax) +82-2-880-7831

Email) psh@snu.ac.kr

**Supplementary Fig. 1. ^1^H-^13^C 2D HSQC for water-soluble extracts from PANC-1 cells and genes affected by external lactate.**

(a) ^1^H-^13^C 2D HSQC spectrum of the water-soluble extracts (left). 1D slice along the carbon dimension of the peaks for alanine (middle) and glutamate (right). (b) Gene ontology enrichment analysis for MCF-7 cells treated with 10 mM lactate. The raw data is from GSE29406 dataset.

**Supplementary Fig. 2. Analysis of ^13^C-carbon incorporation into histone H4 tail using Asp-N and MALDI-TOF.**

(a) The primary sequence of H4 tail and the cleavage site of Asp-N. Theoretical patterns of the MALDI-TOF spectrum from various combinations of H4 methylation and acetylation with Asp-N digestion. Scales are for within-cluster only, and not for inter-cluster comparison. (b) Theoretical isotopomers peak patterns of a peak cluster according to the ^13^C-acetyl incorporation. Left: 100% ^12^C-actyl group; Middle: 100% ^13^C-acetyl group denoted by red dots; Right: overlay of 100% ^12^C, ^13^C, and 50% ^13^C-acetylation.

**Supplementary Fig. 3. Verification of histone modification by lactate metabolism of nuclear LDHA and increase of nuclear LDHA localization in human cancer tissue.**

(a) ^1^H-^13^C 2D HSQC spectrum of pyruvate incubated for overnight and the non-enzymatic formation of acetate. (b) Suppression of non-enzymatic pyruvate degradation into acetate by catalase, as measured by the intensities of the NMR peak on ^1^H-^13^C 2D HSQC spectra. (c) Expression of LDHA observed with western blot in WT and KO cells obtained with CRISPR-Cas9. (d) MALDI-TOF spectra of H4 tail obtained from liver tissue and primary hepatocyte cultured *in vitro*. H4 tail were obtained by the Asp-N treatment. (e) Changes in acetylation of histone H4 by lactate treatment observed by western blot. (f) Cancer and normal adjacent tissues from a pancreatic cancer tissue array (PA241d) were stained with an LDHA antibody. Two regions per tumor and normal tissues were stained as shown on the left. Middle and right images are the magnified ones for the red boxed images on the left.

* Our re-evaluation of the pathology revealed that the patient 3 samples in the purchased array did not contain normal pancreatic cells. Therefore, the patient 3 samples should not be considered for cancer vs. normal comparison.

**Supplementary Fig. 4. Differential nuclear localization of LDHA for various cell lines.**

Nuclear localization of LDHA in pancreatic cancer cell lines as visualized by immunofluorescence. The nucleus was stained with Hoechst (blue) and LDHA with an antibody conjugated with alexa-488 (green).

**Supplementary Table 1. Acetylation of histone H4 altered by lactate observed using ChIP-seq.**

Changes in histone H4 acetylation observed via ChIP-seq after treatment with 0 and 10 mM lactate. ChIP-seq was performed by next generation sequencing (Illumina NovaSeq sequencing (100bp pare-end, 10Gb).

**Supplementary Table 2. Concentrations of metabolites from water-soluble extracts of PANC-1 cells.**

The metabolites were measured and identified with 1D NMR using in-house database and by comparison with standard compounds. The concentrations were calculated with Chenomx software (Edmonton, Alberta, Canada).

**Supplementary Table 3. The ^13^C_3_-lactate incorporation ratio into histone H4 in mouse liver.**

^13^C_3_-lactate at doeses of 0.5 and 1 g/kg were injected into tail veins, and mice were sacrificed at 0, 1, 4 hr time points. Liver tissues were obtained and analyzed for ^13^C labeled histone with MALDI-TOF. Each cluster was normalized by the sum of the total intensity of the cluster, and the incorporation was calculated by the ratio of decrease in the M+0 peak.

**Supplementary Table 4. The ^13^C_3_-lactate incorporation ratio into histone H4 in primary and transformed liver cells.**

^13^C_3_-lactate (10 mM) was treated to the cells overnight, and the H4 incorporation was analyzed with MALDI-TOF spectrum on the Asp-N digest of the H4 tail. The incorporation was calculated as in Supplementary Table 3. * Mean ± standard deviation.

**Supplementary Table 5. Expression of LDHA in the nucleus of human normal and cancer tissues.**

The images and localization annotation of LDHA from immunohistochemistry data in Human protein atlas (proteinatlas.org) were analyzed. The nucleus LDHA information is as annotated in the database. Two different validated antibodies were used for LDHA. The staining intensities and the ratios of the number of samples with nuclear localization to the total number of samples are given.

**Supplementary Table 6. Survival analysis according to the LDHA expression level in pancreatic adenocarcinoma.**

The survival data is from the TCGA pancreas adenocarcinoma cohort. The LDHA high and low groups were divided to give the optimum *p*-value. *HR = Hazard Ratio.

**Supplementary Table 7. LDHA localization in normal and tumor tissues.**

The localization data of LDHA is from the human protein atlas. Antibodies (CAB015336 and CAB069404) were used for LDHA detection. N.D: Not detected; C: cytosol; M: mitochondria; N; Nucleus.

**Supplementary Fig. 1. ^1^H-^13^C 2D HSQC for water-soluble extracts from PANC-1 cells and genes affected by external lactate.**


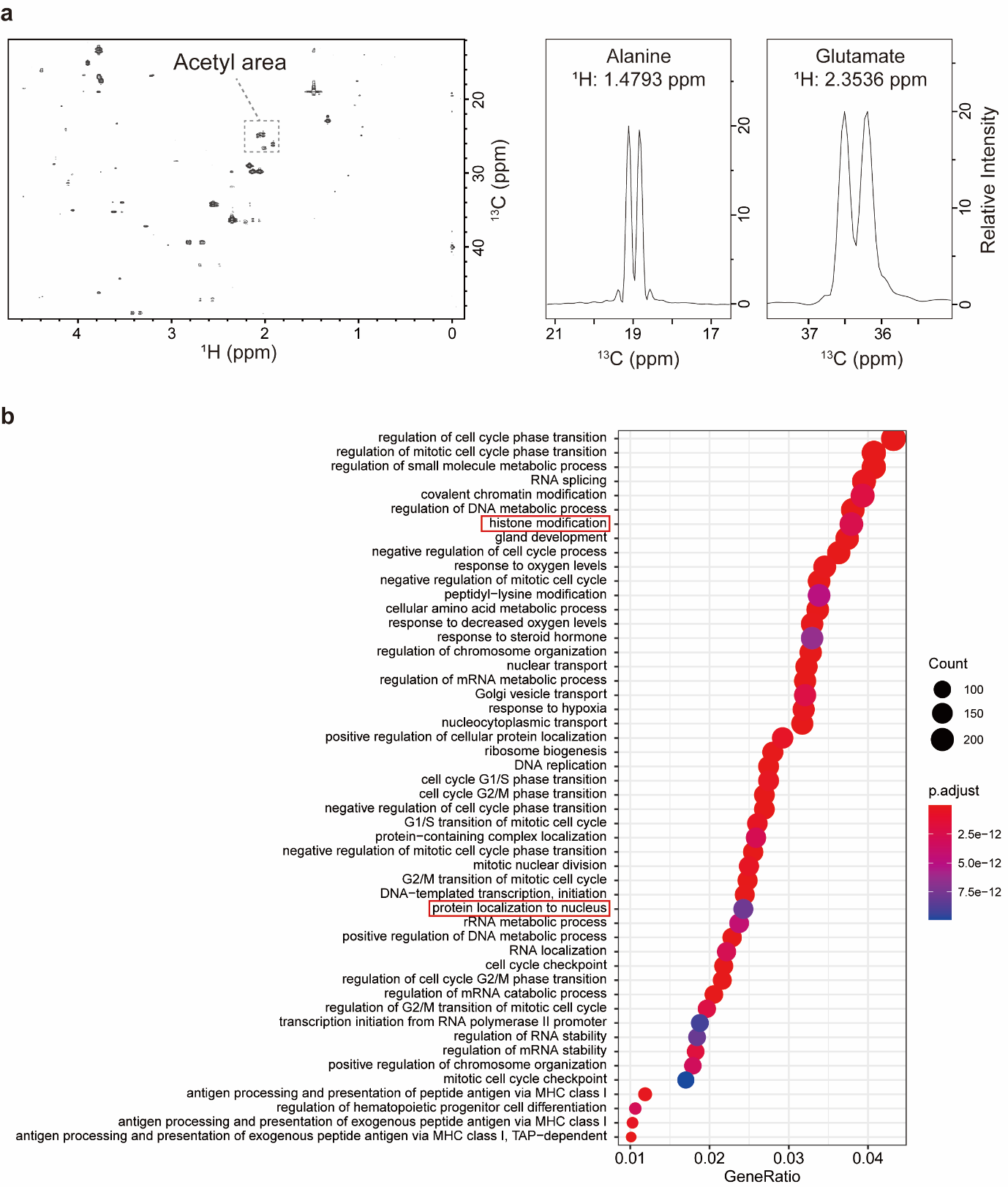


**Supplementary Fig. 2. Analysis of ^13^C-carbon incorporation into histone H4 tail using Asp-N and MALDI-TOF.**

**
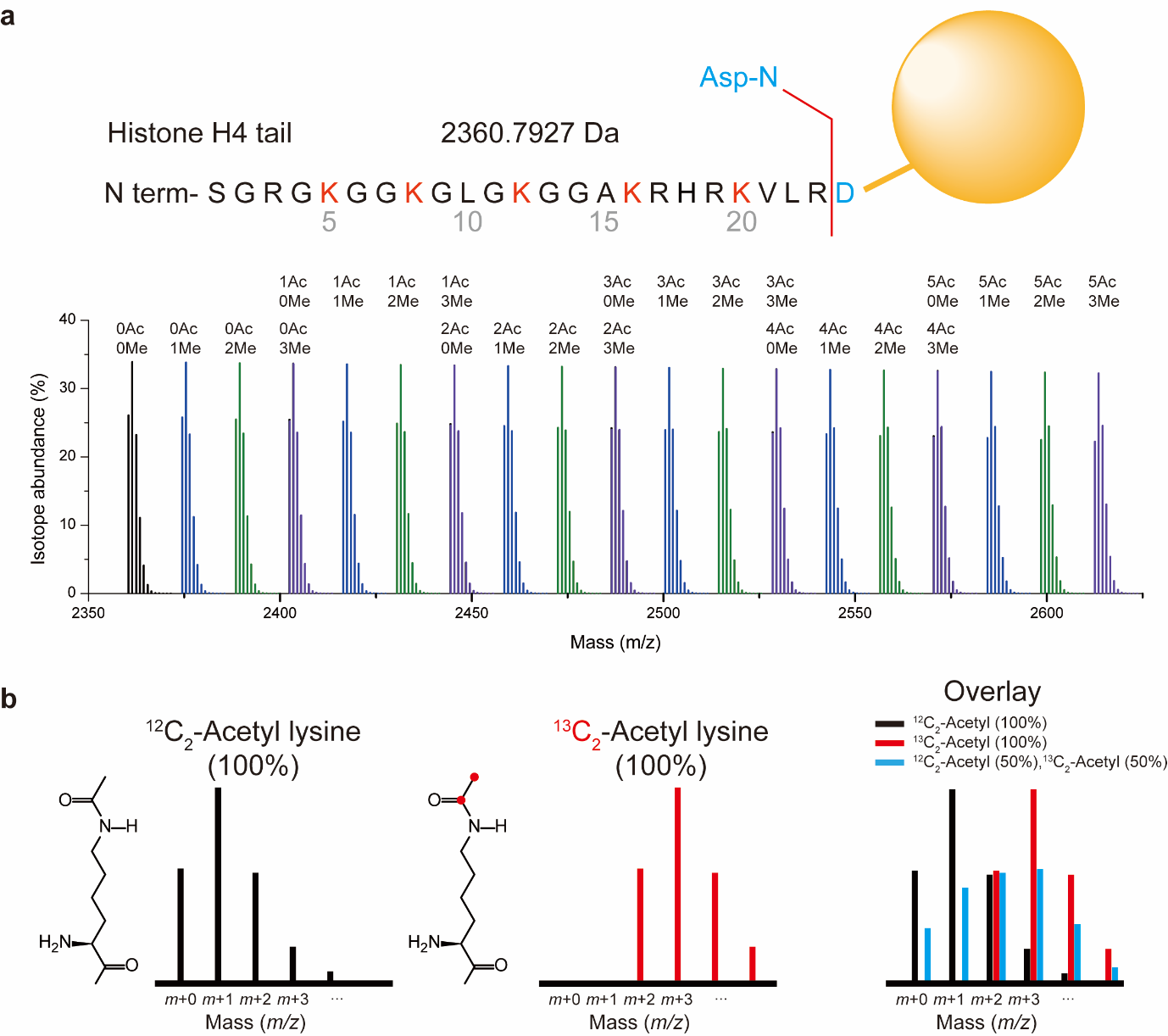
**

**Supplementary Fig. 3. Verification of histone modification by lactate metabolism of nuclear LDHA and increase of nuclear LDHA localization in human cancer tissue.**


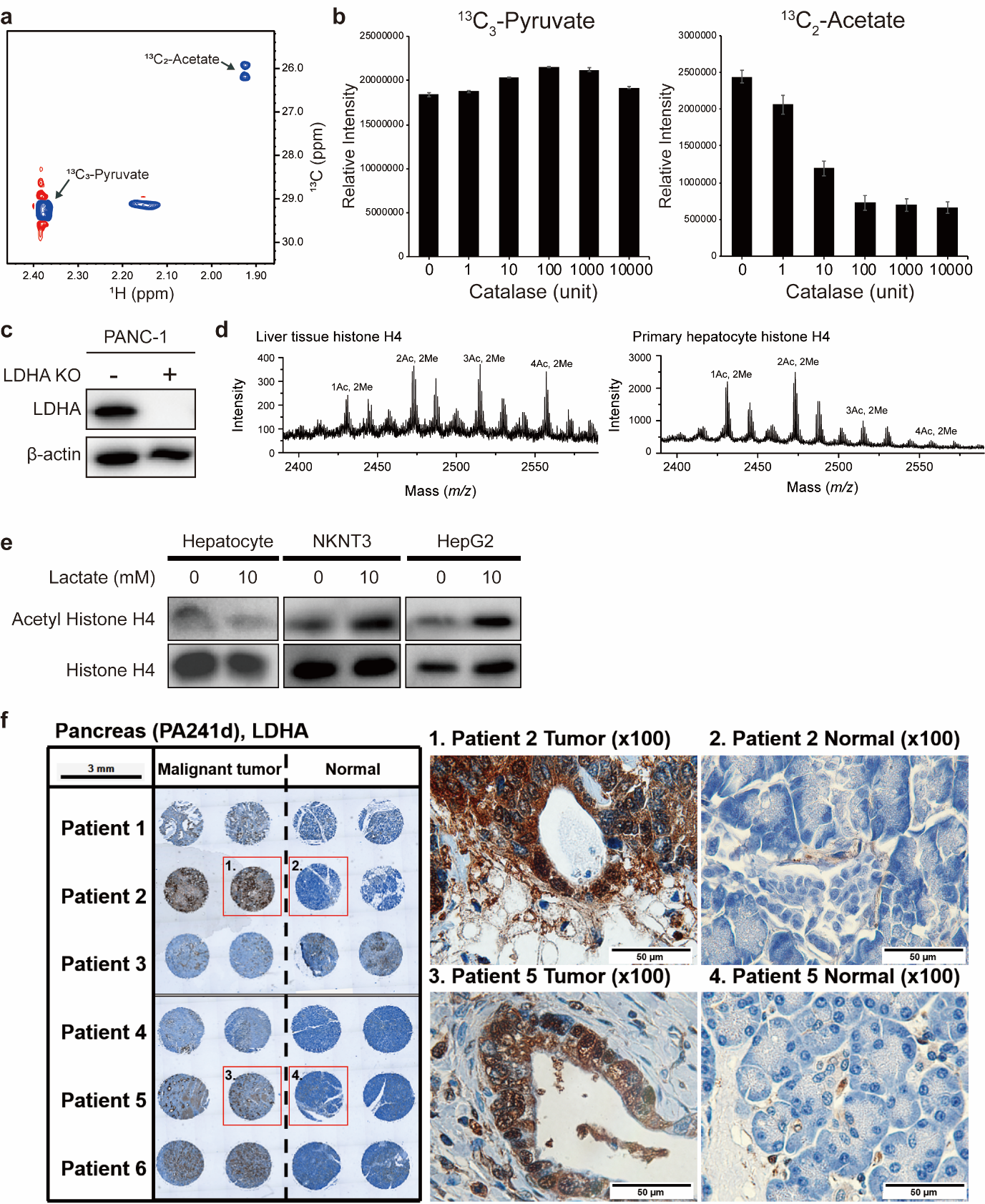


**Supplementary Fig. 4. Differential nuclear localization of LDHA for various cell lines.**

**
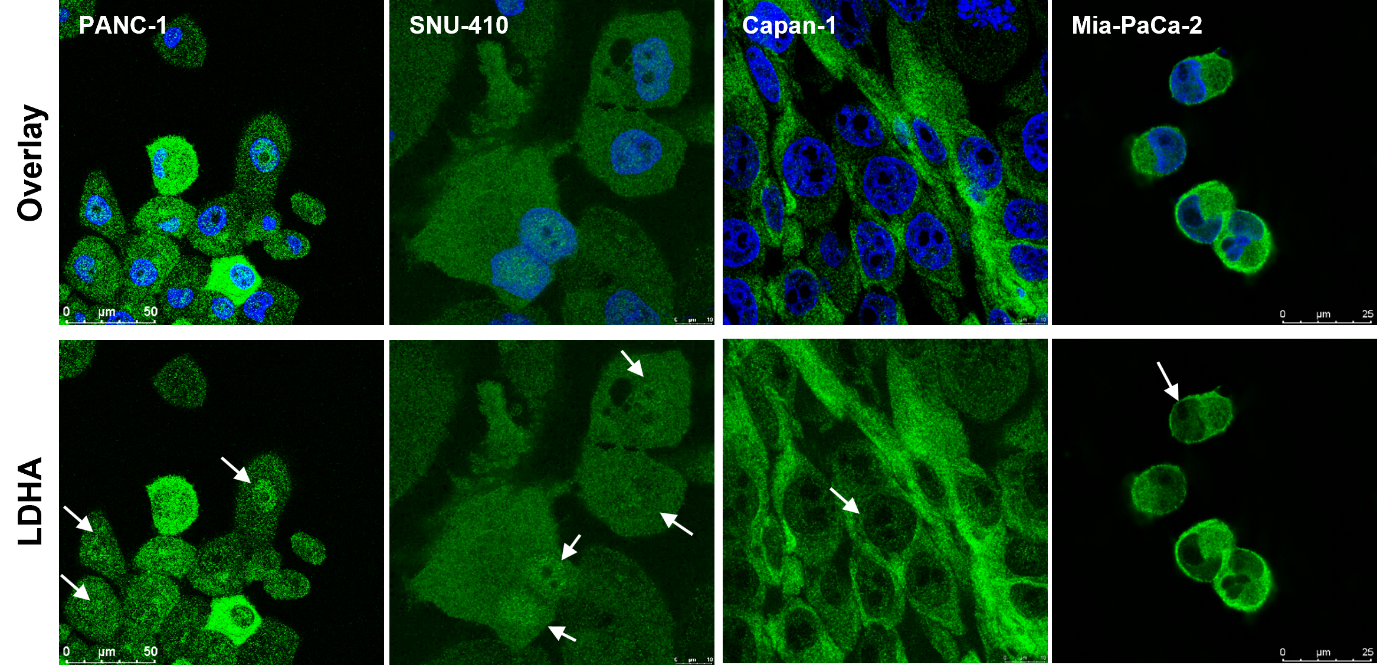
**

**Supplementary Table 1. Acetylation of histone H4 altered by lactate observed using ChIP-seq.**

Supplementary Table 1 was provided the file separately as an Excel.

**Supplementary Table 2. Concentrations of metabolites from water-soluble extracts of PANC-1 cells.**

| Compound Name | Concentration (pM/Cell) | Compound Name | Concentration (pM/Cell) |
| --- | --- | --- | --- |
| myo-inositol | 7.61 | Isoleucine | 0.80 |
| Glutamate | 6.46 | Ethanol | 0.77 |
| O-phosphocholine | 3.89 | Creatine phosphate | 0.67 |
| Glucose | 3.42 | Creatine | 0.67 |
| Glutamine | 2.76 | N-Acetyl-aspartate | 0.63 |
| GSH | 2.54 | Valine | 0.54 |
| Lactate | 2.22 | Alanine | 0.52 |
| Threonine | 1.92 | Phenylalanine | 0.27 |
| Glycine | 1.56 | NAD^+^ | 0.27 |
| Taurine | 1.33 | Pyruvate | 0.25 |
| Proline | 1.31 | Choline | 0.21 |
| Glycero-3-phosphocholine | 1.24 | UDP-N-Acetyl-glucosamine | 0.20 |
| Aspartate | 1.19 | Tyrosine | 0.19 |
| ANP | 0.98 | Acetate | 0.09 |
| Leucine | 0.85 |  |  |

**Supplementary Table 3. The ^13^C_3_-lactate incorporation ratio into histone H4 in mouse liver.**

| ^13^C incorporation  ratio (%) | | 10 mg ^13^C_3_-lactate | | 20 mg ^13^C_3_-lactate | |
| --- | --- | --- | --- | --- | --- |
|  |  | 1 hr | 4 hr | 1 hr | 4 hr |
| 2Me | 2Ac | 4.4±4.2 | 6.2±5.2 | 14.6±4.4 | 34.0±21.3 |
|  | 3Ac | 9.3±1.9 | -0.4±2.4 | 31.4±11.0 | 37.4±7.8 |
|  | 4Ac | -6.7±3.1 | -0.4±3.8 | Overlap | Overlap |

**Supplementary Table 4. The ^13^C_3_-lactate incorporation ratio into histone H4 in primary and transformed liver cells.**

| ^13^C incorporation ratio (%)* | | Primary | NKNT-3 | HepG2 |
| --- | --- | --- | --- | --- |
| 2Me | 1Ac | 7.1±3.2 | 8.9±2.3 | 14.8±2.7 |
|  | 2Ac | 6.5±2.2 | 35.8±1.7 | 47.6±2.7 |
|  | 3Ac | 12.9±6.5 | 46.3±2.7 | 59.7±2.9 |
|  | 4Ac | 16.1±11.8 | 46.2±4.0 | 49.0±4.8 |

**Supplementary Table 5. Expression of LDHA in the nucleus of human normal and cancer tissues.**

| Tissue | LDHA antibody | Intensity | Nucleus LDHA /  Number of patients | Percent |
| --- | --- | --- | --- | --- |
| Cancer liver | CAB015336 | Moderate | 11 / 11 | 100% |
|  | CAB069404 | Strong, Moderate, Weak | 7 / 12 | 58.3% |
| Normal liver | CAB015336 | Moderate | 3 / 3 | 100% |
|  | CAB069404 | Strong | 0 / 3 | 0% |
| Cancer pancreas | CAB015336 | Weak, Moderate | 10 / 10 | 100% |
|  | CAB069404 | Strong | 3 / 8 | 37.5% |
| Normal pancreas | CAB015336 | Weak | 0 / 3 | 0% |
|  | CAB069404 | Moderate | 0 / 3 | 0% |

**Supplementary Table 6. Survival analysis according to the LDHA expression level in pancreatic adenocarcinoma.**

|  | Number of patients | | | Survival Statistics | | |
| --- | --- | --- | --- | --- | --- | --- |
|  | Total | LDHA high | LDHA low | Log-rank *p* | HR* | HR*  (*p*-value) |
| Overall survival | 177 | 43 | 134 | 8.53E-07 | 2.82 | 2.39E-06 |
| Disease-specific survival | 171 | 40 | 131 | 6.52E-07 | 3.18 | 2.37E-06 |
| Disease-free interval | 69 | 20 | 49 | 1.92E-03 | 3.42 | 3.48E-03 |
| Progression-free interval | 177 | 66 | 111 | 4.80E-05 | 2.20 | 7.22E-05 |

**Supplementary Table 7. LDHA localization in normal and tumor tissues.**

| **Normal** | | | | | **Tumor** | | | | |
| --- | --- | --- | --- | --- | --- | --- | --- | --- | --- |
| **Organ** | **N. D** | **C, M** | **C,M,N** | **N** | **Organ** | **N. D** | **C, M** | **C,M,N** | **N** |
| Stomach | 0% | 73% | 27% | 0% | Stomach cancer | 0% | 50% | 50% | 0% |
| Liver | 50% | 25% | 25% | 0% | Liver cancer | 0% | 22% | 78% | 0% |
| Lung | 25% | 25% | 25% | 25% | Lung cancer | 0% | 45% | 55% | 0% |
| Kidney | 0% | 75% | 0% | 25% | Renal cancer | 0% | 22% | 78% | 0% |
| Rectum | 0% | 100% | 0% | 0% | Carcinoid | 25% | 25% | 25% | 25% |
| Colon | 0% | 60% | 40% | 0% | Colorectal cancer | 0% | 57% | 43% | 0% |
| Pancreas | 50% | 50% | 0% | 0% | Pancreatic cancer | 0% | 28% | 67% | 6% |
| Lymph node | 0% | 100% | 0% | 0% | Lymphoma | 9% | 45% | 45% | 0% |
| Thyroid gland | 0% | 100% | 0% | 0% | Thyroid cancer | 0% | 17% | 67% | 17% |
| Ovary | 25% | 75% | 0% | 0% | Ovarian cancer | 4% | 43% | 48% | 4% |
| Breast | 0% | 100% | 0% | 0% | Breast cancer | 0% | 41% | 59% | 0% |
| Cervix | 0% | 25% | 75% | 0% | Cervical cancer | 4% | 30% | 61% | 4% |
| Endometrium | 25% | 50% | 25% | 0% | Endometrial cancer | 0% | 41% | 55% | 5% |
| Brain | 43% | 46% | 11% | 0% | Glioma | 45% | 15% | 35% | 5% |
| Proximal digestive tract | 0% | 12% | 88% | 0% | Head and Neck cancer | 17% | 17% | 67% | 0% |
| Prostate | 40% | 0% | 60% | 0% | Prostate cancer | 24% | 14% | 62% | 0% |
| Testis | 0% | 50% | 50% | 0% | Testis cancer | 33% | 43% | 24% | 0% |
| Skin | 0% | 33% | 67% | 0% | Melanoma | 9% | 39% | 52% | 0% |
|  |  |  |  |  | Skin cancer | 5% | 43% | 48% | 5% |
| Urinary bladder | 67% | 33% | 0% | 0% | Urothelial cancer | 0% | 52% | 48% | 0% |
| **Total** | **17%** | **54%** | **26%** | **3%** | **Total** | **9%** | **34%** | **53%** | **4%** |

N. D: Not detected; C: Cytosol; M: Mitochondria; N: Nucleus.
